# Lysyl oxidases: linking structures and immunity in the tumor microenvironment

**DOI:** 10.1007/s00262-019-02404-x

**Published:** 2019-10-25

**Authors:** Paolo Tenti, Luca Vannucci

**Affiliations:** 1 Laboratory of Immunotherapy, Institute of Microbiology of the Czech Academy of Sciences, Videnska 1083, 142 20 Prague 4, Czech Republic; 2 Faculty of Science, Charles University, Albertov 6, 128 00 Prague, Czech Republic

**Keywords:** Lysyl oxidases, Tumor microenvironment, Immunity, Extracellular matrix structure, TGF-β, CITIM 2019

## Abstract

The lysyl oxidases (LOXs) are a family of enzymes deputed to cross-link collagen and elastin, shaping the structure and strength of the extracellular matrix (ECM). However, many novel “non-canonical” functions, alternative substrates, and regulatory mechanisms have been described and are being continuously elucidated. The activity of LOXs, therefore, appears to be integrated into a complex network of signals regulating many cell functions, including survival/proliferation/differentiation. Among these signaling pathways, TGF-β and PI3K/Akt/mTOR, in particular, cross-talk extensively with each other and with LOXs also initiating complex feedback loops which modulate the activity of LOXs and direct the remodeling of the ECM. A growing body of evidence indicates that LOXs are not only important in the homeostasis of the normal structure of the ECM, but are also implicated in the establishment and maturation of the tumor microenvironment. LOXs’ association with advanced and metastatic cancer is well established; however, there is enough evidence to support a significant role of LOXs in the transformation of normal epithelial cells, in the accelerated tumor development and the induction of invasion of the premalignant epithelium. A better understanding of LOXs and their interactions with the different elements of the tumor immune microenvironment will prove invaluable in the design of novel anti-tumor strategies.

## Introduction

The classically described function of the lysyl oxidases (LOXs) as collagen and elastin cross-linkers could suggest a role restricted to the regulation of the extracellular matrix (ECM). In reality, the activity of LOXs integrates a complex network within the tissue microenvironment (either normal or pathologic) linking bi-directionally the ECM, the immunological components, and the signaling pathways regulating cell survival/proliferation/differentiation. A change in each of these elements propagates to another and LOXs are often involved as key mediators of these changes. The complex cross-talk between these elements of the tissue microenvironment is only partially understood, because the LOXs are involved in a broad spectrum of “non-canonical” functions, such as oxidation of alternative substrates or alternative signaling pathways which only recently have started being elucidated.

We have here reviewed some of these alternatives or recently described pathways and functions, as well as the classical older and most studied ones. Particular importance has been given to the mechanisms commonly dysregulated in cancer and especially relevant to the ECM remodeling and tumor microenvironment (TME) establishment. A special effort has been profuse in trying to illustrate their intricacy and interdependence and proposing some hypothesis on their cancer-related functional aspects.

## LOXs: structure, activation, and physiology

Lysyl oxidase (LOX) family members include five copper-dependent amino-oxidases: LOX, and lysyl oxidase-like 1–4 (LOXL-1, LOXL-2, LOXL-3, and LOXL-4). All of them present a highly conserved carboxy-terminal region containing a cytokine-rich domain and the catalytic domain, which includes a lysyl tyrosyl quinone (LTQ) cofactor and a copper-binding site characterized by a histidine-rich motif [1–3]. The catalytic domain is responsible for the oxidative deamination of the ε-amino groups of lysine and hydroxylysine residues to form highly reactive allysine residues; these tend to condense spontaneously among them or with non-oxidized lysine to form intramolecular and intermolecular covalent bonds (cross-linking). Collagen and elastin are the canonical substrates, and their cross-linking provides mechanical resistance and structural integrity of the ECM [4].

The N-terminal region, except for the signal peptide, is instead poorly conserved. The LOX translated peptide, which has a predicted M.W. of 48 kDa (pre-LOX), is subjected to signal peptide removal and *N*-glycosylation before being secreted extracellularly as the 50 kDa immature form (pro-LOX). After that, the N-terminal 18 kDa lysyl oxidase pro-peptide (LOX-PP) needs to be cleaved by the bone morphogenetic protein-1/pro-collagen C metalloproteinase (BMP-1) to release the active enzyme [5, 6]. Unlike LOX, the immature LOXL-1 presents a proline-rich domain contained in the N-terminal region and has a predicted M.W. of 63 kDa. BMP-1 also activates it; however, the maturation site is not clear, with reports of Western Blotting bands of 66, 55, 41, and 33 kDa following the BMP-1 cleavage [1]. LOXL-2, LOXL-3, and LOXL-4 present even more divergent N-terminal regions, which contain four consecutive scavenger receptor cysteine-rich domains (SRCR) [7]. These LOX family members have a predicted M.W. of 87, 80.3, and 82 kDa, respectively, and in contrast with LOX and LOXL-1, it is uncertain if they require a proteolytic cleavage for their activation [8, 9]. LOXL-2 observed size is around 100 kDa both intracellularly and extracellularly, presumably exceeding the predicted size of 87 kDa because of *N*-glycosylation or other types of post-translational modifications [10]. Several studies have also characterized a 65 kDa extracellular form as a processed LOXL-2 [10, 11]; after the extracellular secretion, in fact, LOXL-2 can be cleaved by a serine protease in the N-terminal region with the consequent removal of the first two SRCR domains. This modification has been shown not to increase LOXL-2 amine oxidase activity with small soluble substrates; however, it is required to stabilize the insoluble scaffold of the collagen IV of the basement membrane. The tridimensional collagen IV scaffold of basement membrane results from both hexameric interactions of two protomers at the C terminus and dodecameric interactions of four protomers at the N terminus. Two protomers interact at the C terminus through the noncollagenous globular C-terminal domain (NC1), while four protomers interact at the N terminus through the 7S domains (7S Dodecamer). Only the 65 kDa form of LOXL-2 can cross-link the 7S Dodecamer and stabilize the collagen IV tridimensional scaffold of the basement membrane [10]. Potential effects of LOXL-2 processing on collagen I and III or other substrates, however, have not been investigated yet.

Based on a phylogenetic analysis of the conserved domains and similarity of the N-terminal region, LOXs can be separated in a first group, including LOX and LOXL-1, and a second group including LOXL-2, LOXL-3, and LOXL-4 [12]. The former group is thought to be phylogenetically newer and preferentially associated with the cross-linking of phylogenetically newer substrates, such as fibrillar collagen (I and III) and elastin; therefore, LOX and LOXL-1 are considered matrix-oriented enzymes, and indeed, they interact with other ECM proteins such as BMP-1, fibronectin, fibulin 4 and 5, and tropoelastin [12]. Being responsible for intrafibrillar and interfibrillar cross-linking, they are fundamental for genesis and maintenance of the fibrillar structure, size, shape, spacing, and mechanical strength [13]. Their function is equally important in the elastogenesis. Experiments in LOX and LOXL-1 knock-out mice showed a predominant role of LOX in the fibrillogenesis and of LOXL-1 in the elastogenesis [14, 15]. LOXL-2, LOXL-3, and LOXL-4, instead, with their SRCR domains phylogenetically old and conserved, might act preferentially as crosslinkers of the basement membrane (collagen IV) and be more firmly in control of the ECM stiffness [16]. Furthermore, the SRCR domains are likely to mediate protein–protein interactions to extend the spectrum of substrates or signaling capabilities of LOXL-2, LOXL-3, and LOXL-4; indeed, SRCR domains are frequently encountered in the structure of the pattern recognition receptors [1, 17].

## LOXs and mechanisms of regulation/dysregulation

It is increasingly recognized that the ECM and its reorganization are of paramount importance in the evolution of both tumor and immune microenvironment, as well for their interaction [18, 19]. LOXs are critical players in the ECM homeostasis, and in fact, their dysregulation is involved in several pathologies, including fibrosis and cancer. Advancing our knowledge on LOXs and their regulation mechanisms is, therefore, paramount to understand the events leading to the TME evolution, both in the early stages (where increasing pro-survival and proliferation stimuli as well as blunted immune surveillance start to manifest), and in more advanced stages, characterized by the appearance of invasive and metastatic phenotypes.

TGF-β can be considered the master regulator of the ECM, modulating the expression of both structural and enzymatic proteins, and this way orchestrating deposition, modification, and degradation of collagen and other ECM components. It is a crucial factor in the maturation of the tumor immuno-microenvironment (TIME): besides its activity on the ECM, TGF-β is a cytokine with a well-recognized role in promoting the differentiation of the T-helper progenitors toward regulatory T cells and is, therefore, thought to be instrumental in blunting the immune surveillance and permitting the tumor development. Furthermore, TGF-β dual role either as a tumor suppressor or as a promoter can be exploited by the tumor by progressively shifting this cytokine activity from prevalently cytostatic in normal epithelial cells to prevalently immune suppressive and pro-carcinogenetic in the TME [20, 21].

In the canonical TGF-β pathway, a family of proteins called Smad (small mother against decapentaplegic) transduces the signals from the TGF-β receptor to the nucleus. TGF-β receptor type I (TGFβRI) phosphorylates Smad2 and Smad3, enabling the binding with the co-factor Smad4 and the formation of active complexes which enter the nucleus. Importantly, Smad4 can bind and activate the Lox promoter [22].

A study in cardiac fibroblasts showed a concomitant upregulation of both LOX, collagen type I and III, and BMP-1 following TGF-β stimulation. LOX upregulation was time and dose-dependent and was abrogated by the inhibition of any of either Smad3, PI3K/Akt (phosphatidylinositol 3-kinase/protein kinase B) or MAPK signaling. PI3K/Akt and MAPK are known to increase TGF-β expression by inducing the activator protein-1 (AP-1) transcription factor. Interestingly, the inhibition of PI3K/Akt and MAPK signaling caused an LOX suppression which was not rescued by TGF-β over-stimulation, suggesting that PI3K/Akt and MAPK effects on LOX go beyond the upregulation of TGF-β. Similarly, PI3K inhibition also abrogated TGF-β-induced upregulation of BMP-1 and collagen [23].

Taken together, these findings suggest that TGF-β orchestrates the organization of the ECM by coordinate transcriptional regulation of LOX and other functionally related proteins. Such process, however, depends on a complex integration of signals; in fact, TGF-β-induced LOX upregulation requires integration of the Smad-dependent pathway with PI3K/Akt and MAPK signaling. Similarly, a PI3K/Akt mechanism has been shown to mediate TGF-β-induced upregulation of collagen and BMP-1 [23, 24].

Smad3 is the main responsible for TGF-β-induced activation of Akt (Smad3 inhibitor SIS3 reduces Akt phosphorylation significantly, although not entirely). While Smad3 increases Akt phosphorylation with a PI3K-dependent mechanism (PI3K inhibitor Wortmannin abrogates the effect), PI3K in return increases Smad3 phosphorylation [23, 25]. This positive feedback loop between Smad3 and PI3K can amplify TGF-β-driven LOX expression by increasing Smad3 phosphorylation and Akt downstream signaling and represents a relevant mechanism of integration between TGF-β and PI3K/Akt signaling.

BMP-1 is the primary activator of at least two LOX family members (LOX and LOXL-1). BMP-1 also cleaves the latent TGF-β-binding protein (LTBP), thus liberating the small latent complex (SLC) from the ECM. The SLC released in the ECM is processed by the matrix metalloproteinase-2 (MMP-2), which removes the latency-associated protein (LAP), this way generating the active form of TGF-β [26]. As TGF-β upregulates both LOX and BMP-1, and the latter activates both LOX and TGF-β, the positive feedback loop between TGF-β activation and BMP-1 upregulation also drives LOX upregulation and activation. At the same time, LOX-induced collagen cross-linking increases the ECM stiffness which (as discussed later) also produce mechanical activation of TGF-β, further fueling the process.

LOX, having a hypoxia-responsive element (HRE) in its promoter, is highly upregulated by the hypoxia-inducible factor-1 (HIF-1), which in turn is increased by LOX enzymatic activity. LOX activity, and in particular its byproduct H_2_O_2_, was shown to activate PI3K/Akt pathway by the increased phosphorylation of 3-phosphoinositide-dependent protein kinase-1 (PDPK-1) and Akt, this way increasing HIF-1α expression at the translational level. No concomitant upregulation at transcriptional level or HIF-1α stabilization was, however, observed [27]. Importantly, Akt/mTOR (Protein kinase B/Mammalian target of rapamycin) and Akt/NF-kB pathways are known to increase HIF-1α expression at the translational and transcriptional levels, respectively [28, 29]. A complex positive feedback loop, therefore, amplifies the signal: active Akt increases HIF-1-dependent LOX expression, while LOX activity, in turn, feeds back Akt activation. LOX, LOXL-2, and LOXL-4 are under direct HIF-1 transcriptional control [27, 30–32]. The Notch pathway and the tumor suppressor LKB1 (Liver kinase B1) also participate to HIF-1-dependent LOX regulation; the former enhances HIF-1 recruitment to the LOX HRE, causing LOX upregulation, while the latter, blocking mTOR/HIF-1 axis, downregulates LOX expression [33, 34].

MAPK signaling is a critical TGF-β non-canonical (i.e., Smad-independent) pathway; TGFβRI engages TRAF6 (TNF receptor associated factor 6) to activate TAK1 (TGF-β-activated kinase 1) which, via different MAPK kinases (MKKs), leads in turn to the activation of the stress-activated kinases c-Jun N-terminal kinase (JNK) and p38 [25, 35, 36]. TGFβRI is also able to activate RAS, which in turn activates JNK and the extracellular signal-regulated protein kinase (ERK) [37, 38]. MAPK activation, either secondary to TGF-β non-canonical pathway or different mechanisms (e.g., hyperactive RAS, NF-kB stimulation, cytokines, and growth factors production), has a fundamental impact on TGF-β signaling. Besides the direct effects on the oncogenes c-Myc and AP-1, MAPKs are also able to lead to non-canonical phosphorylation of Smad2 and Smad3, interfering with the TGF-β canonical pathway. In the canonical TGF-β signaling, TGFβRI causes serine residues phosphorylation at the C terminus of Smad2 and Smad3 (pSmad2C and pSmad3C). On the other hand, MAPKs—as well as other non-canonical pathways, e.g., RhoA/ROCK (Ras homolog family member A/Rho-associated protein kinase) [39]—activate Smad2 and Smad3 by non-canonical phosphorylation of serine and threonine residues in the middle linker region (pSmad2L and pSmad3L) [20, 39–42].

Several studies have shown the association between particular Smad phospho-isoforms (p-isoforms) and specific types of signal [40, 43, 44].

pSmad3C and pSmad2C canonical p-isoforms, prevalent in homeostatic conditions in epithelial cells, transmit a cytostatic signal by c-Myc down-regulation and induction of cyclin-dependent kinases’ (CDKs’) inhibitors [45].

The non-canonical p-isoforms pSmad2L/C and pSmad3L/C—produced intranuclearly by CDKs-dependent phosphorylation of pSmad2C and pSmad3C, following MAPKs activation and c-Myc upregulation—transmit a transient mitogenic and fibrogenic signal inducing proliferation in the epithelial cells and activation and ECM deposition in mesenchymal cells.

In the presence of sustained MAPK activation, the non-canonical p-isoforms pSmad3L and a cytoplasmically produced form of pSmad2L/C are produced constitutively. pSmad3L transmits a sustained mitogenic and fibrogenic signal by c-Myc upregulation inducing proliferation in the epithelial cells and activation and ECM deposition in mesenchymal cells [39, 45, 46]. pSmad2L/C is produced in the cytoplasm by TGFβRI-mediated phosphorylation of the cytoplasmically retained pSmad2L. The phosphorylation of pSmad2L to pSmad2L/C enables the nuclear translocation and the transmission of a sustained invasive and fibrogenic signal leading to upregulation of plasminogen activator inhibitor-1 (PAI-1) and MMP-9 [40, 43]. PAI-1 increases the ECM deposition and modifies the degree of cellular adhesion contributing to cell migration [47]. MMPs enhance cell invasion through the ECM proteins’ degradation, which causes the release of active cleavage fragments and the opening of spaces, where cells can invade [40].

It is likely that different non-canonical signalings, from both non-Smad pathways and non-canonical Smad p-isoforms, integrate each other, and work synergistically to produce proliferative, invasive, and fibrogenic signals [45, 48].

The effects of non-canonical signaling on the activity of LOXs, to our knowledge, have not been studied. We can speculate that different types of Smad p-isoforms and TGF-β signals influence differently the expression and activation of LOXs. It is plausible, for example, that a fibrogenic signal might be able to enhance greatly the activity of LOXs, while a cytostatic signal, on the other end, is likely to increase their activity to a smaller extent, if at all.

TGF-β is often dysregulated in cancer. The shift from canonical to non-canonical TGF-β signaling (discussed above) represents a significant mechanism of dysregulation. Other mechanisms, which typically increase in frequency with the tumor progression, include inactivating mutations of signaling cascade and decreased expression of TGFβRI or TGFβRII [49–51]. Despite the progressive termination of its cytostatic/apoptotic signal, TGF-β tends to be over-expressed in cancer, with a possible variability depending on the stage of evolution [52]. The autocrine production by neoplastic cells is often involved, but TGF-β is also produced by activated fibroblasts, regulatory T cells, tumor-associated macrophages, and myeloid-derived suppressive cells, which are part of the TIME [53, 54].

**Fig. 1:**
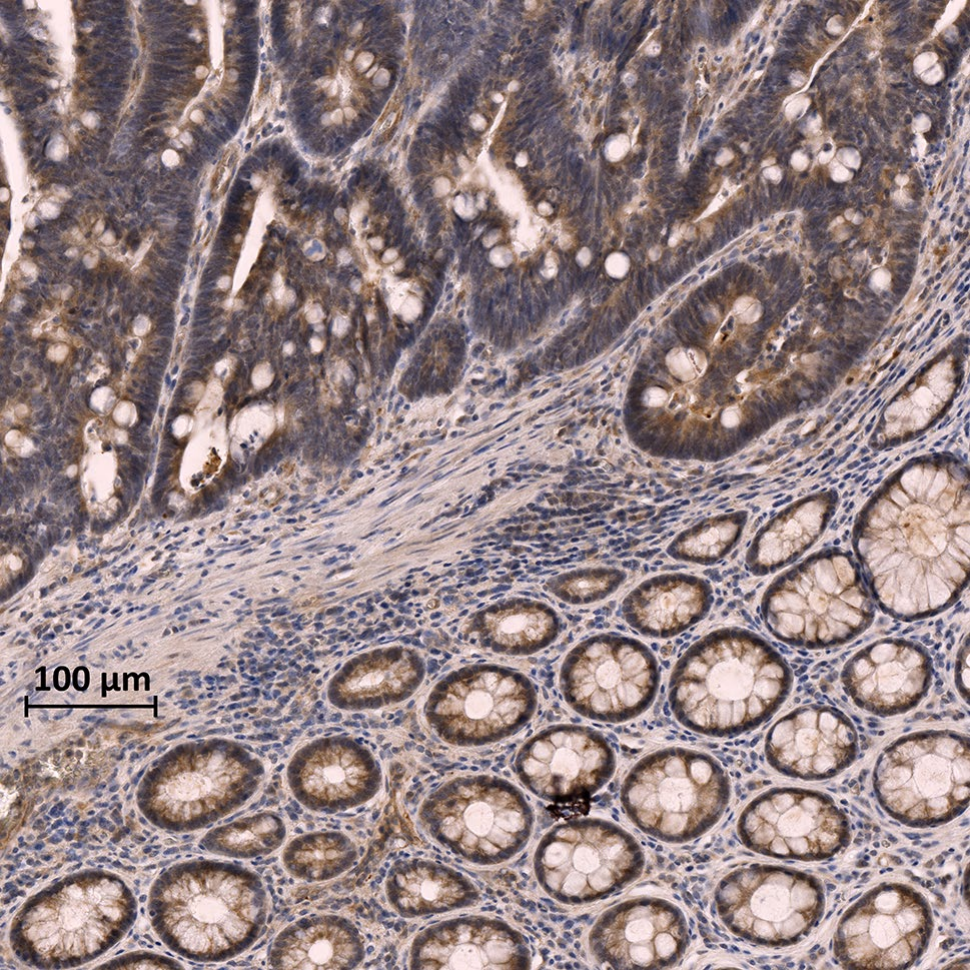
LOXL-2 expression in AOM-induced rat colon cancer. Large positivity to LOXL-2 expression in the tumor tissue (upper part of the image) in comparison with the non-neoplastic mucosa (lower part of the image). Phosphatase anti-phosphatase immunohistochemistry with anti-LOXL-2 primary antibodies developed with DAB (brown color). *AOM* azoxymethane, *DAB* 3,3′-diaminobenzidine

Many reports indicate that one or more LOX family members are also over-expressed in invasive and metastatic cancers [30, 55–57]. The mechanisms of LOXs’ overexpression in cancer, to our knowledge, have not been elucidated. Activated stromal cells recruited in the TME are likely involved; however, tumor cells might also contribute to the overexpression of LOXs. TGF-β and LOXs need to be tightly regulated to maintain the ECM homeostasis in normal conditions, but neoplastic cells might evade the regulatory mechanisms and produce LOXs constitutively (Fig. 1). In any case, as TGF-β is often over-expressed and dysregulated in cancer, the increase of LOXs is likely, at least in part, TGF-β driven. TGF-β orchestrates the organization of the ECM and increases the expression of LOXs in normal conditions; therefore, it is plausible that TGF-β dysregulation might associate with the overexpression of LOXs, and that their increased activity might be critical for the reorganization of the ECM and the establishment and maturation of the TIME.

In normal conditions, TGF-β increases the expression of LOX and LOX, in turn, acts in a negative feedback to downregulate TGF-β [58, 59]. It has been shown a dose-dependent binding between LOX and TGF-β, with the binding sites situated in the mature forms (as both full length and mature forms of LOX and TGF-β can bind). Such interaction, localized in the ECM, is associated with suppression of Smad3 phosphorylation; the effect is rescued by beta-aminopropionitrile (BAPN), but is unaffected by catalase, suggesting that LOX direct enzymatic activity, rather than its byproduct H_2_O_2_, suppresses the TGF-β signaling. Given LOX preference for basic substrates with pI > 8, it is thought that the mature TGF-β molecule is targeted at the basic lysine-rich C terminus, and inactivated by oxidative deamination of lysine residues [58, 60].

In vivo experiments with LOX^−/−^ knock-out mice showed significant upregulation (~ 30%) of some TGF-β target genes: SerpinE1 (encoding for PAI-1), CTGF (Connective Tissue Growth Factor), and Col1a2 (Collagen type I alpha 2 chain), confirming that LOX modulates TGF-β signaling [59].

Recently, it has been shown that active LOX binds to the high temperature requirement A serine peptidase-1 (HTRA1), which is a secreted trimeric protease able to degrade TGF-β [61–63]. LOX enzymatic activity induces HTRA1 higher order multimerization, which is required for its activation [61, 64].

LOX activity, therefore, seems able to promote TGF-β degradation both directly, by oxidative deamination at the C terminus of TGF-β, and indirectly, by the activation of the high-temperature requirement A serine peptidase 1 (HTRA1).

**Fig. 2:**
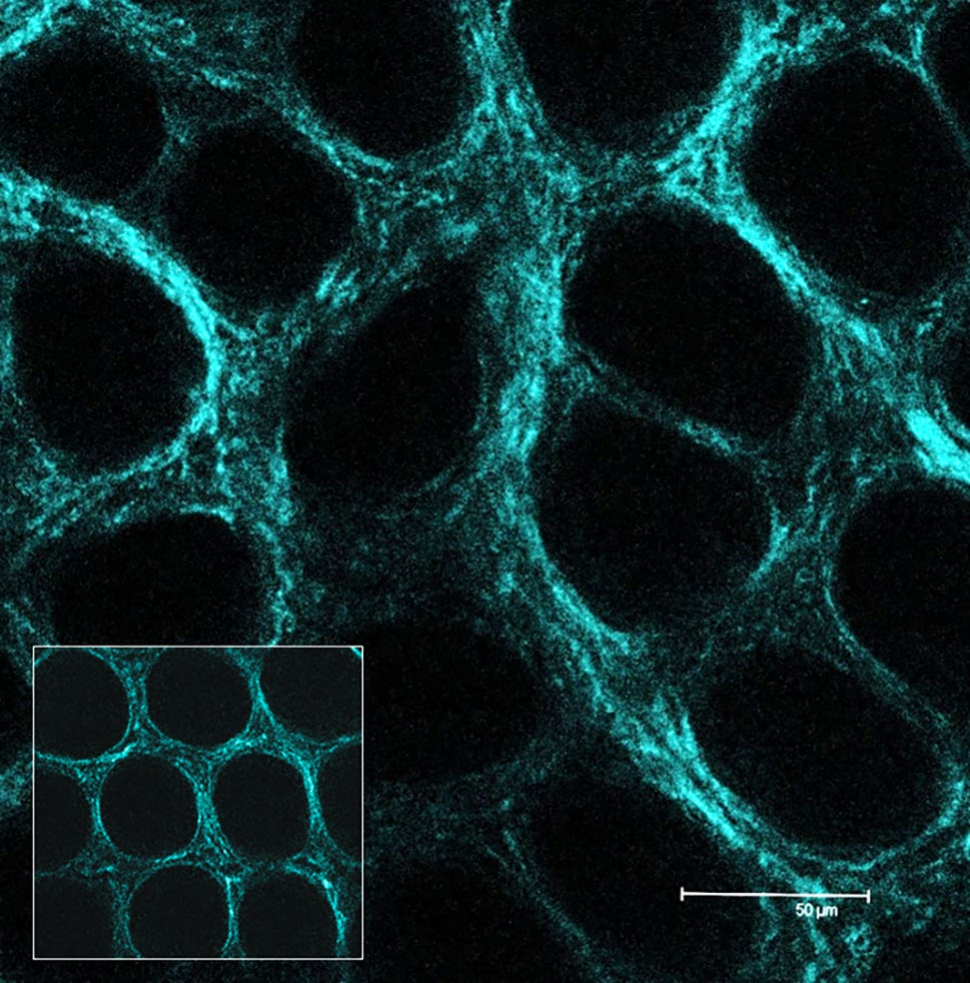
Collagen scaffold structure in the rat colon mucosa. Evidence of increased density and initial alteration of the general architecture symmetry at 1 month after induction of carcinogenesis by AOM. In the square: aspect of the colon mucosa collagen scaffold in healthy rat. 2-photon laser scanning confocal imaging, SHG, ×63 original magnification. *AOM* azoxymethane, *SHG* second harmonic generation

Myofibroblast contraction generates mechanical stress on the ECM, including stretching of the large latent complex (LLC), with the release of TGF-β from LAP. The LLC is anchored to the ECM through the LTBP, while LAP binds to the myofibroblast through the extracellular domain of transmembrane proteins, i.e., integrins. Integrins intracellular domain binds to α-SMA (α-smooth muscle actin) positive stress fibers, which are part of the myofibroblasts cytoskeleton. As a result of α-SMA/myosin interaction, the myofibroblasts contract, and the mechanical tension is propagated from the stress fibers to the LAP via the integrin bound. The entity of LLC stretching and of TGF-β release increases with the stiffening of the ECM; in fact, it is required an ECM which resists the myofibroblast contraction to cause efficient LLC stretching [18, 19, 65]. While there is evidence of LOX acting in a negative feedback loop to promote TGF-β degradation, on the other hand, LOX family members, as the primary regulators of the ECM maturation and stiffening, can increase the release of active TGF-β. We can hypothesize that the negative feedback exerted by LOXs is effective in normal conditions, but as dysregulation of TGF-β-signaling manifests in tumor tissue, an increase in ECM deposition and LOXs also occurs; as a consequence, the ECM stiffness also increases leading to additional release of TGF-β from the extracellular deposit and further dysregulation, thus sustaining a vicious circle. In normal conditions, the balance among TGF-β signaling, ECM deposition, and LOX-mediated collagen cross-linking must be tightly regulated to maintain the tissue homeostasis. However, these interactions can be differently affected by the TME at different steps of its evolution (Fig. 2).

Other described transcription factors regulating LOX at the promoter level includes GATA-binding protein 3 (GATA-3)—which is also associated with T-helper 2 polarization—and the pro-survival transcription factor forkhead box M1b (FoxM1b), often over-expressed in cancer. GATA-3 suppresses LOX through the promoter methylation, while FoxM1b enhances its expression by the direct promoter binding [66, 67].

The above discussion highlights the importance of chronic inflammation in increasing the activity of LOXs. In fact, TGF-β signaling, NF-kB activation (secondary to oxidative stress, growth factors, and inflammatory cytokines such as TNF-α and IL-1), MAPK activation, and HIF-1α upregulation/stabilization are all stress and inflammation response mechanisms which are engaged in extensive cross-talking with LOXs. Given the increased level of one or another LOX family member in many types of cancer, it is strongly suggested a link between inflammation, LOXs, and cancer development.

## LOXs and the TME

Most of the studies on LOXs have focused on LOX and, more recently, on LOXL-2. As the enzymatic activity of LOX family members is thought to be similar, disentangling the differences among the different members has proved challenging and further researches are necessary. LOX-PP, the 162-amino-acid pro-peptide domain of pro-LOX, is considered an oncosuppressor, as it decreases the RAS oncogene signaling by the interaction with c-Raf and the c-Raf chaperone Hsp70 to reduce MAPK/ERK activation [68–70]. Recently, recombinant LOX-PP has been shown to sensitize prostate cancer cells to apoptosis through the nuclear translocation and interaction with the nuclear DNA repair regulator MRE11 at DNA repair foci [71]. Despite this anti-tumor role documented for LOX-PP, and the reduced levels of some LOXs in a minority of cancers, the mature LOX, as well as other LOX family members, has a predominant pro-neoplastic activity. The LOXs increase in invasive and metastatic cancer, and their high expression is correlated with poor survival [30, 55–57]. There is increasing evidence of the importance of LOXs in driving tumor growth and progression; in particular, many studies indicate that the HIF-1/LOXs’ axis is a crucial mechanism in driving the tumor cell proliferation, the epithelial–mesenchymal transition (EMT), the formation of pre-metastatic niches, and the cancer cells migration and invasion [27, 56, 72–75].

It is not clear how a family of enzymes acting on the ECM can induce intracellular signaling to drive tumor progression. The increase in stiffness of the ECM, promoting focal adhesions formation and integrins activation, is the most studied mechanism. The β-integrins (INTβ)—specifically INTβ1, INTβ3, and INTβ4—have an essential role as mechanosensor and transducers of the ECM stiffness. INTβ activation leads to SRC kinase recruitment and SRC-dependent phosphorylation of a focal adhesion kinase (FAK) [73, 76, 77]. Phosphorylated FAK can activate several signaling pathways, including RAS/MAPK and Rho/ROCK, the latter leading to activation/remodeling of the actin cytoskeleton and the acquisition of a migratory phenotype [78]. Active FAK can enhance ErbB2-induced triggering of PI3K/Akt signaling in mammary epithelial cells stimulated by the epidermal growth factor (EGF); however, it is not able to increase Akt directly in unstimulated cells [73]. The mechanisms behind such enhancing effect have not been elucidated; we can speculate that FAK activation might increase the expression or the surface retention of ErbB2, and possibly other related receptors of the EGFR family. It is not known if other receptors are also enhanced by FAK; if so, the ECM stiffness could modulate an even broader range of signals.

Recently, a mechanism of surface “trapping” of the EGFR has been shown to depend on LOX-mediated degradation of TGF-β and to be instead independent of the ECM stiffness and SRC phosphorylation. LOX-mediated degradation of TGF-β (previously reviewed) reduces the suppressive effect of TGF-β signaling on matrilin2 (MATN2), a protein containing ten EGF-like domain. Consequently, MATN2 accumulates and through its EGF-like domains binds EGFR, causing the surface “trapping” of the receptor, i.e., its surface retention. The resulting increased that the availability of EGFR boosts the EGF signaling leading to Akt activation, which in turn drives cell proliferation [61].

LOXs can be produced by different cell types, including epithelial cells, endothelial cells, and activated fibroblasts.

Tumor-secreted LOXL-2 has been described to activate stroma fibroblasts (to α-SMA + myofibroblasts) and vascular smooth muscle cells through the H_2_O_2_-mediated activation of FAK/SRC and ErbB2/Erk2 signaling [79, 80]. LOXL-2 has also been shown to promote fibroblast activation and invasion of the ECM through an INTβ3-dependent but SRC-independent activation of FAK/Akt signaling [81].

Activated stromal cells, in turn, produce LOXL-2 along with chemokines and growth factors such as VEGF, platelet-derived growth factor (PDGF), and fibroblast growth factor (FGF). Therefore, tumor-secreted LOXs act in a positive feedback loop to activate stromal cells to produce further LOXs and growth factors, thus driving the TME remodeling and the tumor progression [81].

It is noteworthy that in vitro experiments with LOXs inhibitors showed a prominent role of LOXL-2 versus LOX in tumor growth, collagen cross-linking, angiogenesis, and fibroblast activation [82]. LOXL-2 expression has also been linked to upregulation of tissue inhibitor of metalloproteinase-1 (TIMP-1) and MMP-9. TIMP-1 promotes resistance to apoptosis, cell proliferation, and metastasis through mechanisms which enhance FAK, Akt, and MAPK phosphorylation and are probably independent of the MMP inhibition [83].

Recently, the hydrogen peroxide-inducible clone-5 (Hic-5) was found over-expressed in cancer-associated fibroblasts (CAFs), but not cancer cells. Hic-5 resulted necessary for the generation of a tumor-permissive microenvironment; in fact, the gene knock-out completely prevented the cancer development in an azoxymethane-induced colorectal cancer mice model. Cancer-related cytokines such as TGF-β, PDGF, IL-1β, and stromal cell-derived factor-1 (SDF-1) were found able to induce fibroblast activation and acquisition of a CAF phenotype including Hic-5 high expression. In turn, Hic-5 accumulated inside the nucleus and induced LOX and type-1 collagen expression. This evidence suggested that TGF-β, PDGF, IL-1β, and SDF-1 induce a Hic-5-rich CAF phenotype which drives the overexpression of LOX and collagen to increase the ECM stiffness and generate a tumor-permissive stroma [84].

During the EMT, immobile transformed cells lose their epithelial polarization and inter-cellular adhesion properties to acquire a migratory and invasive mesenchymal-like phenotype. Characteristic biomarkers of these functional modifications are the down-regulation of E-cadherin (a component of adherens junctions) and the upregulation of α-SMA (a marker of cytoskeletal reorganization), vimentin, and N-cadherin (mesenchymal markers). Many survival/proliferation signals are involved in EMT, including hypoxia, Akt/Snail, WNT/β-Catenin, Notch, and Smad/non-Smad TGF-β signaling, regulating the expression of the so-called EMT transcription factors (EMT-TFs). One of the most studied EMT-TFs is Snail, known for repressing E-cadherin directly at the promoter level [85]. LOXL-2 and LOXL-3 have been shown to stabilize Snail-1 (this way downregulating the E-cadherin) through a protective modification of Snail-1 on the N-terminal SNAG domain. The modified domain cannot be phosphorylated by the glycogen synthase kinase-3 beta (GSK3B); therefore, Snail-1 ubiquitination and targeting for proteasomal degradation are prevented [86]. LOXL-2 has also been reported to interact with E47 EMT-TF, cooperating in the repression of the E-cadherin promoter [87]. LOXs are often critically involved in the EMT: either by direct upregulation of EMT-TFs, or indirectly by an upstream process, or by complex combinations of them. LOX-mediated stiffening of the ECM leads to the release of active TGF-β as well as activation of FAK/SRC, both of them contributing to the loss of adhesion and acquisition of a motile phenotype [78]. LOXs enhance Snail-1 activity partly by direct Snail-1 stabilization and partly by upregulation at translational level of HIF-1, which in turn promotes Snail-1 activation, besides upregulating other EMT-TFs such as ZEB1 [31, 88, 89]. FoxM1b drives EMT by Akt-mediated block of GSK3B and consequent Snail-1 stabilization; at the same time, it upregulates at promoter level LOX and LOXL-2, which also stabilize Snail-1 and activate Akt, besides driving EMT by the increase in stiffness of the ECM [67].

Hypoxia-driven production and secretion of LOXs are deemed a fundamental component of the tumor secretome leading to the formation at secondary sites of permissive niches, where the environment is prepared for the metastatic colonization (pre-metastatic niche). In this process, LOX-dependent cross-linking of type IV collagen in the basement membrane provides the signal for bone marrow CD11b + myeloid cells’ recruitment and chemotaxis [32, 90].

LOXL-2 activity is regarded as essential in the ECM remodeling required for the neo-angiogenesis, as it regulates the sprouting of neo-vessel through its accumulation in the endothelial cells and the assembling of type IV collagen in their basement membrane [16]. Both LOXs and VEGF are hypoxia signature genes, suggesting their cooperative role under HIF control.

## Final remarks and conclusions

Experimental data have focused prevalently on LOX, and more recently, to a lesser extent, on LOXL-2. LOX family members are likely to present a partially overlapping activity, but also distinct functions, specific for each member and types of tissues and physiopathological conditions. Such a difference in activities is also documented by recent publications. The involvement of one or more members of LOXs (in the active form or as the pro-peptide) was evaluated in different cancers and conditions: experimental ornithine decarboxylase- and RAS-transformed mouse fibroblasts and human melanoma cells, renal and bladder, and gastric cancers, respectively [91–93]. LOXs showed variability of expression during tumor development, with the possibility of exerting either promotion or even inhibition effects depending on the type of LOX expressed, the tumor type, and the microenvironment. In a prostate cancer model, BAPN-induced inhibition of LOXs resulted in tumor suppressive effects when treatment was initiated before the tumor implantation and tumor-promoting effects when treatment was initiated after the tumor establishment [94]. These findings strongly suggest the importance of studying LOXs in the specific microenvironment of the tissue, where the tumor arises, according to the TME modifications during its establishment and evolution.

Until now, the research of inhibitors of LOXs has principally focused on LTQ-directed molecules, which bind the LTQ cofactor irreversibly inhibiting their enzymatic activity. These molecules are usually primary amines, like in the case of BAPN, and inhibit LOXs specifically (unlike the copper chelators, e.g., disulfiram); however, they are frequently not selective for a specific LOX family member [95, 96]. BAPN, the prototypic and most utilized inhibitor of LOXs, is usually considered a pan-inhibitor, although several conflicting studies report a negligible activity of BAPN on one or another LOX family member; therefore, it is necessary to exercise caution when interpreting the effects of non-selective inhibitors such as BAPN [97]. The development of selective inhibitors would help significantly to disentangle the effects of one LOX family member from another, but the process is still hindered by the unavailability of the crystal structure for each of these molecules. Cell-permeable inhibitors would also be beneficial, allowing to discriminate between intracellular and extracellular functions of LOXs.

Recently, a monoclonal antibody has been employed successfully in the selective inhibition of LOXL-2. This approach proved effective in contrasting the development of the TME in in vivo cancer models, reducing fibroblast activation, growth factors production, and TGF-β signaling. It also significantly reduced the tumor burden, outperforming the treatment with BAPN [82].

The unavailability of valuable genetically modified animal models for each LOX family member is another limit for these studies. In the case of LOX, the production of gene knock-out animals resulted in 100% lethality (end of gestation or perinatal period), associated with severe cardiovascular malformations [4, 14, 98]. LOXL-2 knock-out animals also suffer 50% perinatal mortality and a high incidence of severe cardiovascular defects [99]. These catastrophic effects highlight the fundamental role of LOXs in the tissue morphogenesis and stroma maturation.

A majority of studies have focused on the effects of LOXs on the tumor progression and development of metastatic disease. However, there is also strong evidence supporting a significant role of LOXs in the transformation of normal epithelial cells, in the accelerated tumor development and the induction of invasion of the premalignant epithelium [61, 73, 79].

The networking with the TGF-β signaling is also intriguing for the possible links between these molecules, the tumor structure development, and the immune microenvironment progressive organization during the tumorigenesis and, later on, the formation of the metastatic niches.

The recent discoveries of several new substrates and alternative catalytic and non-catalytic functions of LOXs (e.g., as transcription or co-transcription factors) show a complex and multifaceted involvement in the biology and immunology of the TME. Thus, we believe that a better understanding of the structure and function of these molecules will prove extremely valuable to further enlighten the tumor-immunity interactions and to design novel anti-tumor strategies targeting the TIME development and evolution.

## Abbreviations

AP-1: Activator protein-1
BAPN: Beta-aminopropionitrile
BMP-1: Bone morphogenetic protein-1
CAF: Cancer-associated fibroblasts
JNK: c-Jun N-terminal kinase
CDKs: Cyclin-dependent kinases
EGF: Epidermal growth factor
EMT: Epithelial–mesenchymal transition
EMT-TFs: Epithelial–mesenchymal transition transcription factors
ECM: Extracellular matrix
ERK: Extracellular signal-regulated protein kinase
FAK: Focal adhesion kinase
FoxM1b: Forkhead box M1b
GATA-3: GATA-binding protein 3
GSK3B: Glycogen synthase kinase-3 beta
HTRA1: High-temperature requirement A serine peptidase 1
Hic-5: Hydrogen peroxide-inducible clone-5
HIF-1: Hypoxia-inducible factor-1
HRE: Hypoxia-responsive element
LLC: Large latent complex
LAP: Latency-associated protein
LTBP: Latent TGF-β-binding protein
LOX: Lysyl oxidase
LOX-PP: Lysyl oxidase pro-peptide
LOXL: Lysyl oxidase-like
LOXs: Lysyl oxidases
LTQ: Lysyl tyrosyl quinone
mTOR: Mammalian target of rapamycin
MATN2: Matrilin2
MMP: Matrix metalloproteinase
p-isoforms: Phospho-isoforms
PAI-1: Plasminogen activator inhibitor-1
PDGF: Platelet-derived growth factor
Akt: Protein kinase B
RhoA/ROCK: Ras homolog family member A/Rho-associated protein kinase
SRCR: Scavenger receptor cysteine-rich domains
pSmad2C: Smad2 phosphorylated at the C terminus
pSmad2L/C: Smad2 phosphorylated in the middle linker region and at the C terminus
pSmad2L: Smad2 phosphorylated in the middle linker region
pSmad3C: Smad3 phosphorylated at the C terminus
pSmad3L: Smad3 phosphorylated in the middle linker region
pSmad3L/C: Smad3 phosphorylated in the middle linker region and at the C terminus
SLC: Small latent complex
Smad: Small mother against decapentaplegic
SDF-1: Stromal cell-derived factor-1
TGFβRI: TGF-β receptor type I
TIMP-1: Tissue inhibitor of metalloproteinase-1
TIME: Tumor immuno-microenvironment
TME: Tumor microenvironment
INTβ: β-Integrin
α-SMA: α-Smooth muscle actin
